# Differential Adaptation in Azimuth and Elevation to Acute Monaural Spatial Hearing after Training with Visual Feedback

**DOI:** 10.1523/ENEURO.0219-19.2019

**Published:** 2019-10-30

**Authors:** Bahram Zonooz, A. John Van Opstal

**Affiliations:** Donders Centre for Neuroscience, Department of Biophysics, Radboud University, Nijmegen 6525 AJ, The Netherlands

**Keywords:** auditory system, human, models, perceptual learning, plasticity, sound localization

## Abstract

Sound localization in the horizontal plane (azimuth) relies mainly on binaural difference cues in sound level and arrival time. Blocking one ear will perturb these cues, and may strongly affect azimuth performance of the listener. However, single-sided deaf listeners, as well as acutely single-sided plugged normal-hearing subjects, often use a combination of (ambiguous) monaural head-shadow cues, impoverished binaural level-difference cues, and (veridical, but limited) pinna- and head-related spectral cues to estimate source azimuth. To what extent listeners can adjust the relative contributions of these different cues is unknown, as the mechanisms underlying adaptive processes to acute monauralization are still unclear. By providing visual feedback during a brief training session with a high-pass (HP) filtered sound at a fixed sound level, we investigated the ability of listeners to adapt to their erroneous sound-localization percepts. We show that acutely plugged listeners rapidly adjusted the relative contributions of perceived sound level, and the spectral and distorted binaural cues, to improve their localization performance in azimuth also for different sound levels and locations than those experienced during training. Interestingly, our results also show that this acute cue-reweighting led to poorer localization performance in elevation, which was in line with the acoustic–spatial information provided during training. We conclude that the human auditory system rapidly readjusts the weighting of all relevant localization cues, to adequately respond to the demands of the current acoustic environment, even if the adjustments may hamper veridical localization performance in the real world.

## Significance Statement

Plugging one ear in normal-hearing listeners disrupts the robust binaural difference cues, leading to a dramatic impairment of sound-localization accuracy in the horizontal plane. We trained plugged listeners to localize sounds in the horizontal plane through visual feedback about the true sound location. We show that the auditory system rapidly reweights the different binaural and monaural localization cues to improve performance in azimuth. Quite unexpectedly, we also found a strong degradation of localization performance in the elevation direction, even on the intact hearing side, which resulted from the training. We conclude that the auditory system rapidly adapts to current acoustic situations to optimize localization performance, even if these changes reduce performance for other acoustic environments, like encountered in daily life.

## Introduction

Sound localization relies on the processing of acoustic cues that result from the interaction of sound waves with head, torso, and pinnae. For directions in the horizontal plane (azimuth), the human brain relies on interaural time differences (ITDs) for frequencies ≤1.5 kHz, and on interaural level differences (ILDs) for higher frequencies (≥3 kHz). The ITDs and ILDs do not specify the elevation angle (up-down, front-back) of sound sources. The latter relies on idiosyncratic spectral-shape cues from direction-dependent acoustic reflections and refraction within the pinna cavities, described by head-related transfer functions (HRTFs). This broadband (BB) spectral mechanism defines a unique monaural elevation cue for frequencies ≥3–4 kHz (for review, see Blauert, 1997; Van Opstal, 2016).

The existence of seemingly independent mechanisms to extract the azimuth and elevation coordinates has some interesting corollaries that are unique to the auditory system. For example, localization performance in elevation can be heavily perturbed without a deterioration of azimuth localization, e.g., by inserting binaural pinna molds (Hofman et al., 1998; Morimoto, 2001; Hofman and Van Opstal, 2003; Carlile, 2014), by adding background noise (Zwiers et al., 2001), or by varying sound levels, spectra, and sound durations (Butler, 1987; Hofman and Van Opstal, 1998; MacPherson and Middlebrooks, 2000; Vliegen and Van Opstal, 2004).

Under monaural hearing conditions, the binaural time- and level-differences are heavily perturbed or absent, which severely hampers azimuth localization (Oldfield and Parker, 1986; Moore et al., 1999; Kacelnik et al., 2006; Van Wanrooij and Van Opstal, 2007; Kumpik et al., 2010; Agterberg et al., 2012; Keating and King, 2013; Keating et al., 2016; Kumpik and King, 2019). Four additional cues could subserve azimuth localization under perturbed binaural hearing: (1) the level-related head-shadow effect (HSE), (2) weakened binaural level differences, (3) the spectral cues from the hearing ear, and (4) low-pass (LP) filtering by the head (Oldfield and Parker, 1986; Van Wanrooij and Van Opstal, 2007; Kumpik and King, 2019). Note that the monaural head-shadow cue is ambiguous, as a loud sound at the perturbed side may be perceived just as loud as a soft sound at the hearing side. A similar ambiguity holds for the head’s LP filter (Van Wanrooij and Van Opstal, 2004, 2007). Therefore, the veridical location of the sound source cannot be specified by these monaural cues alone (Van Opstal, 2016). Yet, in familiar environments, or sounds with known properties, monaural listeners could use the HSE in combination with these priors to better estimate their location (Van Wanrooij and Van Opstal, 2007; Carlile, 2014; Van Opstal, 2016; Kumpik and King, 2019).

Although a monaural plug attenuates high frequencies by 30–50 dB, low-frequency ITDs may pass unobstructed, while for loud sounds, some binaural level differences may survive, albeit biased toward the hearing ear. Indeed, individuals with severe conductive hearing loss still use weak binaural level differences to localize azimuth (Agterberg et al., 2012). Clearly, this potential cue is not available for single-sided deaf listeners (Van Wanrooij and Van Opstal, 2004).

Under monaural hearing, pinna cues from the hearing ear may contribute to localize azimuth (Van Wanrooij and Van Opstal, 2004, 2007). Indeed, the auditory system of ferrets and humans can compensate for monaural occlusion by using spectral cues from the good ear to perceived azimuth (Van Wanrooij and Van Opstal, 2007; Keating et al., 2016; for review, see Kumpik and King, 2019). Studies on listeners with severe conductive hearing loss, single-sided deafness, and normal-hearing but acutely plugged listeners support this idea, but reported considerable idiosyncratic variability as to how much these listeners used spectral cues for azimuth localization (Agterberg et al., 2012; Van Wanrooij and Van Opstal, 2004, 2007).

Training with feedback may further enhance and speed-up sound-localization performance under perturbed hearing. For example, monaurally plugged listeners improve spatial hearing in azimuth through audiovisual training (Shinn-Cunningham et al., 1998; Strelnikov et al., 2011; Mendonça et al., 2013; Mendonça, 2014). The auditory system can also reweight acoustic spectral contributions to localize elevation when repeatedly exposed to sounds with only weak spectral cues (Zonooz et al., 2019).

Here, we assessed localization performance in azimuth and elevation of normal-hearing listeners after acute monaural plugging. We studied the effect of repeated exposure to a single high-pass (HP) filtered sound of fixed intensity at a limited number of locations in the horizontal plane, by providing visual feedback. We assessed whether listeners learned to remap the different acoustic cues to improve localization performance, and whether they generalized their learned behavior to other sounds presented across the two-dimensional frontal hemifield.

## Materials and Methods

### Participants

Eight binaural listeners (S1, S3–S8: ages 23–27, and S2: age 61; four females) participated in the free-field sound-localization experiments. All, except for S7, were naive regarding the purpose of the study. The inexperienced subjects were given a brief practice session to get acquainted with the setup and localization paradigms, and to gain stable localization performance to standard BB Gaussian white-noise stimuli. Subjects S1, and S3–S8 had normal hearing (within 20-dB hearing level) in both ears, as assessed with a standard audiometric test from 0.25 up to 8 kHz. Subject S2 (female) had binaural high-frequency hearing loss of 25–30 dB at 6 kHz, and 40–50 dB at 8 kHz. Consequently, the elevation responses of S2 deviated substantially from the other subjects (see Results).

### Ethics statement

Human subjects were recruited at the Radboud University. The local Ethics Committee of the Faculty of Social Sciences of the Radboud University (protocol number ECSW 2016-2208-41) approved the experimental procedures, as they concerned non-invasive observational experiments with healthy adult human subjects. All experimental protocols adhered to the relevant guidelines and ethical procedures. Before their participation in the experiments, subjects gave their full written consent.

### Experimental setup

During the experiments, subjects sat comfortably in a chair in the center of a completely dark, sound-attenuated room (length × width × height: 3.6× 3 × 3 m). The walls of the room were covered with black foam that prevented echoes for frequencies exceeding 500 Hz. The background noise level in the room was ∼30-dB SPL. Target locations and head-movement responses were transformed to double-polar coordinates (Knudsen and Konishi, 1979). In this system, azimuth, α, is defined as the angle between the sound source or response location, the center of the head, and the midsagittal plane, and elevation, ε, is defined as the angle between the sound source, the center of the head, and the horizontal plane. The origin of the coordinate system corresponds to the straight-ahead speaker location. Head movements were recorded with the magnetic search-coil induction technique (Robinson, 1963). To that end, the participant wore a lightweight (150 g) “helmet” consisting of two perpendicular 4-cm-wide straps that could be adjusted to fit around the participant’s head without interfering with the ears. On top of this helmet, a small coil was attached. From the left side of the helmet, a 40-cm-long, thin, aluminum rod protruded forward with a dim (0.15 Cd/m^2^) red LED attached to its end, which could be positioned in front of the listener’s eyes, and served as an eye-fixed head pointer for the perceived sound locations. Two orthogonal pairs of 3 × 3 m coils were attached to the edges of the room to generate the horizontal (60 kHz) and vertical (80 kHz) magnetic fields. The head-coil signals were amplified and demodulated (Remmel Labs), after being LP filtered at 150 Hz (custom-built 4th order Butterworth filter) before being stored on hard disk at a sampling rate of 500 Hz per channel for off-line analysis.

### Auditory stimuli

Acoustic stimuli were digitally generated using Tucker-Davis Technologies (TDT) System III hardware, with a TDT DA1 16-bit digital-to-analog converter (48.828 125-Hz sampling rate). A TDT PA4 programmable attenuator-controlled sound level, after which the stimuli were passed to the TDT HB6 buffer and finally to one of the speakers in the experimental room. All acoustic stimuli were derived from a standard Gaussian white noise stimulus, which had 5-ms sine-squared onset and offset ramps. This BB GWN control stimulus had a flat amplitude characteristic within 2 dB (uncorrected) between 0.2 and 20 kHz (Zonooz et al., 2019) and a duration of 150 ms.

**Figure 1.**
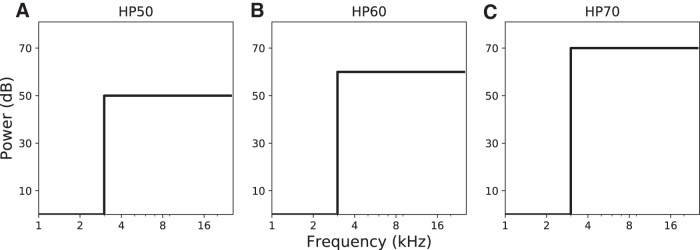
Schematized power spectra of the sound stimuli used in the pre-adaptation/post-adaptation experiment. Stimuli were derived from a GWN control stimulus by removing all frequencies below 3 kHz (HP), at (***A***) 50-dB SPL (A-weighted), (***B***) 60 dBA, or (***C***) 70 dBA.

The three types of stimuli were presented during the control experiments on the first day. BB, LP, and HP contained the frequencies from 0.2 to 20 kHz, all frequencies up to 3.0 kHz and the frequencies above 3.0 kHz, respectively. On the second day of the experiment, which included the adaptation session, only the HP stimuli were chosen, as by focusing on the HP stimuli we excluded the ITD contribution to azimuth sound localization (Fig. 1). Absolute free-field sound levels were measured at the position of the listener’s head with a calibrated sound amplifier and microphone (Brüel and Kjaer).

### Experimental paradigms

#### Calibration

Each experimental session started with a calibration experiment to establish the mapping parameters of the coil signals to known target locations. Head-position data for the calibration procedure were obtained by instructing the listener to make an accurate head movement while redirecting the dim LED in front of the eyes from the central fixation LED to each of 58 peripheral LEDs, which were illuminated as soon as the fixation point extinguished. The 58 fixation points and raw head-position signals thus obtained were used to train two three-layer neural networks (one for azimuth, one for elevation) that served to calibrate the head-position data, using the Bayesian regularization implementation of the back-propagation algorithm (MATLAB; version 15, Neural Networks Toolbox) to avoid overfitting (Pedregosa et al., 2011).

**Figure 2.**
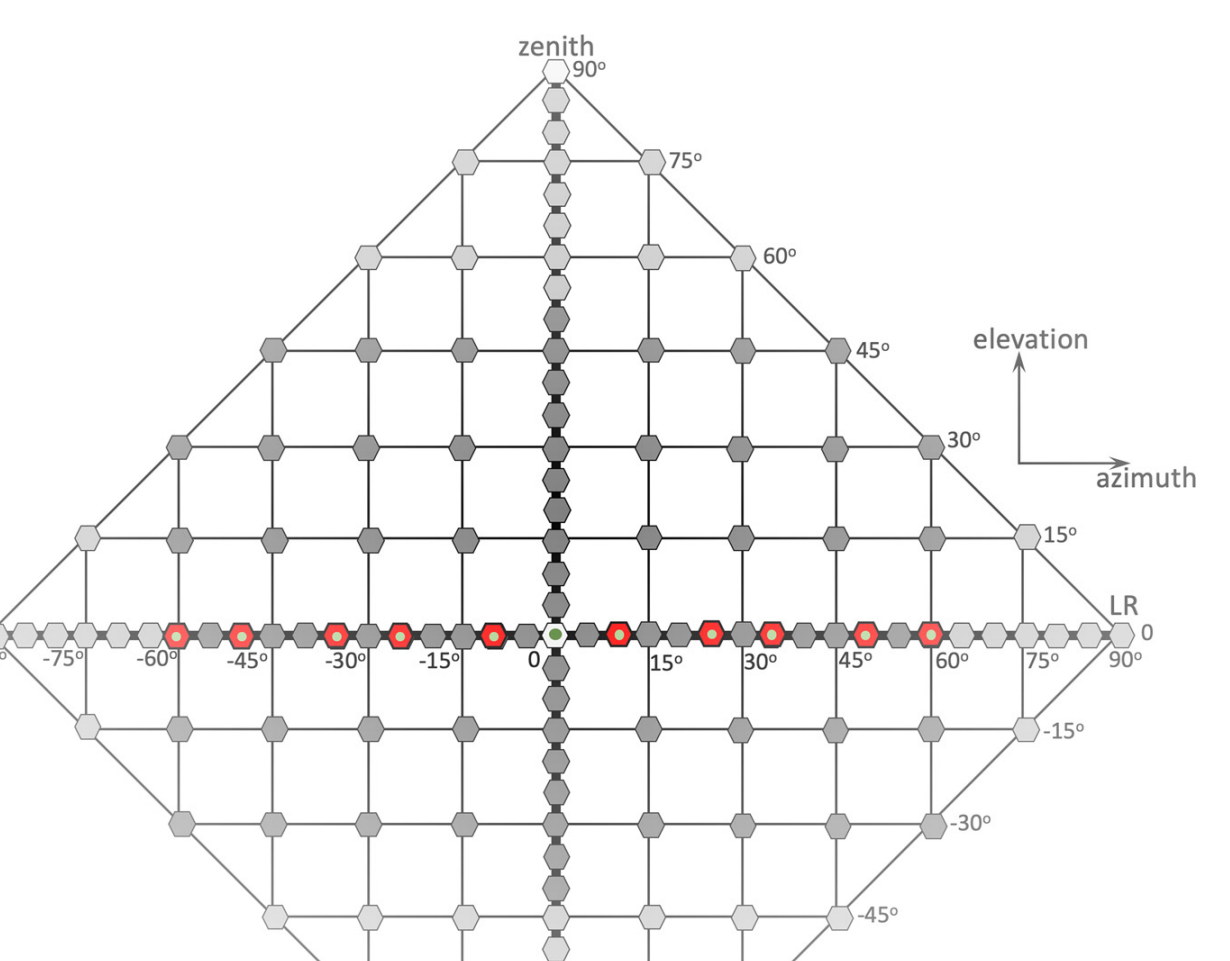
Distribution of sound-source locations, as used in the different experimental paradigms, projected in a flattened Cartesian azimuth-elevation coordinate grid. Note that speakers were attached to a spherical frame, and that in the double-pole azimuth-elevation coordinate system the sum of azimuth and elevation angles can never exceed 90° (outer diamond-shaped boundary). The training targets were located on the azimuth plane, and are indicated in red. They were presented with visual feedback (green dot) at the end of each trial. The pre-adaptation and post-adaptation test targets (red and dark gray) were distributed across the frontal hemifield, and were pseudo-randomly selected for azimuth in [–60,60]°, and for elevation in [–40,+50]°, not including the training targets. In the control experiment of day 1, selected speaker locations were confined to [–80,+80]° for azimuth, and [–40,+50]° for elevation. LL, lateral left; LR, lateral right. The central speaker at (0,0)° and the speaker at the zenith were not used.

In each sound-localization experiment, the listener started a trial by fixating the central LED (azimuth and elevation both at 0°; Fig. 2). After a pseudo-random period between 1.5–2.0 s, this LED was extinguished, and an auditory stimulus was presented 400 ms later. The listener was asked to redirect the head by pointing the dim LED at the end of the aluminum rod to the perceived location of the sound stimulus, as fast and as accurately as possible.

#### Plugging

To heavily perturb the acoustic input to the right ear, we followed the procedures described in Van Wanrooij and Van Opstal (2007). Plugs were made by filling the ear canal with a rubber casting material (Otoform Otoplastik–K/c; Dreve). Earlier measurements in our lab indicated that the precisely fitting plug attenuated high-frequency sounds (>2 kHz) by at least 25 dB. Low frequencies (up to ∼1.5 kHz) were attenuated by ∼20 dB. To ensure further monaural attenuation, and to eliminate any potential spectral cues from the plugged ear, an additional headphone muff was positioned over the plugged ear (Agterberg et al., 2012). Note that although the plug-and-muff hearing condition perturbed the binaural level cues substantially, there could still be some remnant binaural hearing for low frequencies (based on ITD processing), and even some highly perturbed ILDs for (part of the) high frequencies, especially for the loudest sound levels (60 and 70 dBA).

#### Control session

The sound-localization experiments were divided into the two experimental days. The subjects performed the localization control experiment on the first day. This experiment contained 300 trails with BB, LP, and HP stimuli, and were presented at randomly selected locations that ranged from [–80,+80]° in azimuth, and from [–40,+50]° in elevation (Fig. 2). The presented stimuli varied in intensity; sound levels of HP stimuli varied between 45- and 70-dB SPL (A-weighted) in 5-dB increments, sound levels of LP stimuli as well as BB stimuli were either 50 or 65 dBA (HP: 6 different sound levels, 30 locations, in total: 180 trials, and HP, BB each two different sound levels, 30 locations, in total 120 trials). The control experiment served to establish the subject’s pre-adaptation localization abilities, and to verify the effect of sound level on the monaural listeners’ localization performance, before the adaptation experiment. That is, we chose the sound level for which they had developed no prior knowledge (monauralized subjects were unable to localize it accurately). The subjects participated twice in the control experiment, unplugged and plugged. The results were used to verify whether they were indeed normal-hearing and that the plug had a detrimental effect on their localization performance. The pre-adaptation, training, and post-adaptation experiments were performed on a second recording day.

#### Training

In the training experiment, subjects localized the HP stimuli of 60 dBA, presented at 10 fixed locations in the azimuth plane (+60°, +48°, +36°, +24°, +12°, –12°, –24°, –36°, –48°, –60°), at an elevation of 0°. After the sound was presented, and the subject had made the localization response, a green LED in the center of the speaker was illuminated for a duration of 1500 ms. The subject was required to make a subsequent head-orienting response to the location of the LED; this procedure ensured that the subject had access to error signals related to programming a corrective response, immediately after the initial sound-localization estimate. The training experiment consisted of 500 trials in which every location was presented 50 times in pseudo-random order.

#### Test sessions

The pre-adaptation and post-adaptation test experiments contained the same 180 trials, with three types of stimuli: HP50, HP60, and HP70 sounds. Stimuli were presented at pseudo-randomly selected locations in the 2D frontal hemifield, ranging from [–60,+60]° in azimuth, and from [–40,+50]° in elevation (Fig. 2, dark-gray). Note that the test set of stimuli did not include the ten sound locations used during the training. Listeners performed the post-adaptation experiment twice, once with one ear plugged, and once unplugged (both ears free).

### Data analysis

A custom-written MATLAB script automatically detected head saccades in the calibrated data by using a preset velocity criterion (15°/s) for saccade onset and offset. Detected saccades were visually inspected for errors, and manually corrected if necessary, without having access to stimulus information. We analyzed the responses for each participant, separately for the different stimulus types, by determining the optimal linear fits for the stimulus–response relationships for the azimuth and elevation components:

(1) $$R_{\alpha }=a+b\cdot T_{\alpha }\,\;and{\;R}_{\varepsilon }=c+d\cdot T_{\varepsilon },$$

by minimizing the least-squares error, using the Scikit-learn library (Pedregosa et al., 2011). $R_{\alpha }$ and $R_{\varepsilon }$ are the azimuth and elevation response components, and $T_{\alpha }$ and $T_{\varepsilon }$ are the azimuth and elevation coordinates of the target. Fit parameters, a and c, are the response biases (offsets; in degrees), whereas b and d are the response gains (slopes, dimensionless) for the azimuth and elevation response components, respectively. Note that an ideal localizer should yield gains of 1.0, and offsets of 0.0°. We also calculated Pearson’s linear correlation coefficient, *r*, the coefficient of determination, *r* ^2^, the mean absolute residual error (SD around the fitted line), and the mean absolute localization error for each fit.

To determine to what extent the acute monaural listener makes use of the ambiguous HSE and/or the true source location (presumably through distorted weak binaural cues, or spectral cues, see Introduction) to localize sound sources, we also analyzed our data through multiple linear regression. To that end, we evaluated the relative, normalized contributions of sound level and stimulus azimuth to the subject’s azimuth localization response in the following way:

(2) R^α=p⋅I^prox+q⋅T^α⁢ where⁢ z^≡z−μzσz,

Here, ${\hat{R}}_{\alpha },{\hat{I}}_{prox},$ and ${\hat{T}}_{\alpha }$ are the dimensionless *z*-scores for the response, proximal sound level, and target values, respectively, with *μ_z_* the mean, and *σ_z_* the SD of variable z. In this way, the contributions of sound level and sound location can be directly compared, although they are expressed in different units, and may cover very different numerical ranges. The dimensionless partial correlation coefficients, *p* and *q*, quantify the relative contributions of sound level and target azimuth, respectively, to the measured response. A perfect localizer would yield *p* = 0 and *q* = 1, indicating that the localization response is not affected by variations in perceived sound level, and fully determined by changes in source location. On the other hand, if *p* = 1 and *q* = 0 the responses are entirely determined by the HSE.

The proximal sound level, ${\hat{I}}_{prox}$, was calculated as the perceived intensity at the free ear, by using the following approximation:

(3) $${\hat{I}}_{prox}\left(T_{\alpha }\right)=I_{snd}+HSE\cdot \sin\left(\frac{\pi \cdot T_{\alpha }}{180}\right)dB.$$

Here, *I_snd_* is the actual free-field sound level (in dBA) at the position of the head, and the sine function approximates the HSE and ear-canal amplification for a broad-band sound (we took HSE = 10 dB, following Van Wanrooij and Van Opstal, 2004).

For the elevation responses, we extended the multiple regression analysis in the following way:

(4) $${\hat{R}}_{\varepsilon }=p\cdot {\hat{I}}_{prox}+q\cdot {\hat{T}}_{\alpha }+s\cdot {\hat{T}}_{\varepsilon }.$$

Here, the elevation response was considered to potentially depend on proximal sound level, the true target’s azimuth location, and the true target’s elevation angle. For an ideal localizer, the partial correlations should yield [*p,q,s*] = [0,0,1].

## Results

### Azimuth responses

#### Normal hearing

**Figure 3.**
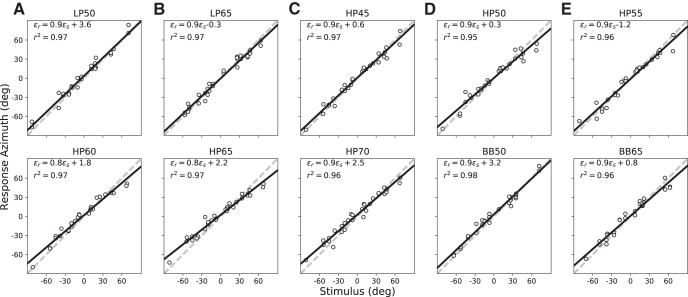
(***A***–***E***) Control results for normal hearing: data from listener S3 in azimuth for the ten control stimuli (LP, HP, and BB at different intensities). Linear regressions (Eq. 1) were performed on the azimuth components of the stimulus–response relations (each point corresponds to a single trial). Responses were highly accurate, as gains and biases were very close to their optimal values of 1.0 and 0.0, respectively.

All listeners (*N* = 8) were first subjected to two control sound-localization experiments, in which they responded with rapid goal-directed head-orienting movements to ten different sound stimuli presented across the frontal hemifield. The normal-hearing localization results in azimuth for participant S3 to these stimuli are shown in Figure 3. Localization performance for all ten stimulus types (LP, HP, and BB noise bursts) in the azimuth plane were near-optimal, irrespective of sound level (45- to 70-dB SPL A-weighted): they exhibited high accuracy, as response gains (Eq. 1) were close to one, and biases close to 0°, with little variability, as evidenced by high *r* ^2^ values for the linear fits (>0.95).

#### Control (plugged)

**Figure 4.**
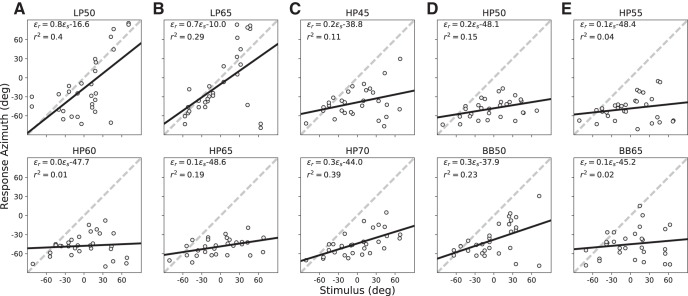
(***A***–***E***) Control responses, right-ear plugged (results for S3). Same format as Figure 3. Responses were highly inaccurate, as the gains and biases deviated substantially from their optimal values of 1.0 and 0.0, respectively. Note that low-frequency ITDs could still be used with the plug/muff, as the response gain is still quite high; yet, variability in the responses is considerably higher than for normal hearing.

When the binaural cues were corrupted after right-ear plugging, S3 was no longer able to localize the stimuli in the horizontal plane (Fig. 4). Although the response gains for the LP sounds remained relatively high (∼0.7), the response variability was considerably higher than for normal-hearing (*r* ^2^ < 0.4). The strongest effects of the plug were obtained for the HP and BB sounds. Responses to these stimuli had a strong leftward (negative) bias toward the hearing ear (typically exceeding –40°), very low response gains (between 0.1 and 0.3), and considerable variability (low correlations). Yet, the response gains for each stimulus were not zero, suggesting that the listener still had some percept of changes in azimuth, possibly due to a combination of monaural spectral cues and highly attenuated binaural level differences.

#### Pre-training (plugged)

**Figure 5.**
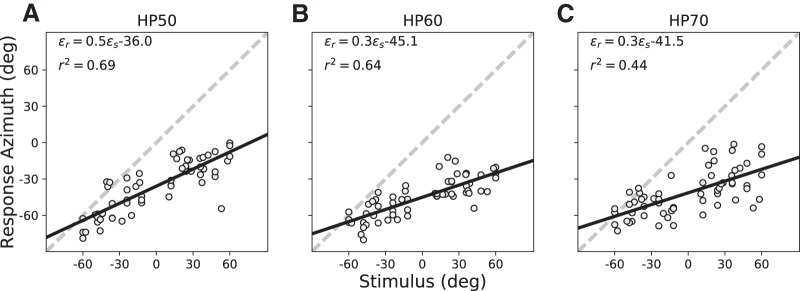
(***A***–***C***) Pre-adaptation localization results for subject S3 in azimuth for the three test HP stimuli with the right ear plugged. Responses were highly inaccurate, as the gains and biases deviated substantially from their optimal values of 1.0 and 0.0, respectively. However, the slopes of the regression lines differed significantly from zero, indicating that the stimuli contained some azimuth-dependent localization cues.

In the pre-training experiment on the second recording day, we first measured the localization performance for three HP filtered stimuli at different levels (HP50, HP60, and HP70), presented across the two-dimensional frontal hemifield. Results for the stimulus-response relationships of the azimuth components for representative listener S3, with the right ear plugged, are shown in Figure 5. The regression data indicate the low precision and accuracy with which this listener responded to these sounds (low gain, large leftward bias, and large variability, when compared to the unplugged condition; compare Fig. 3). Note that the HP60 and HP70 stimuli yielded larger response biases (>45° and >41°, respectively) than the low-intensity HP50 sound (–36°), although the relation between bias and sound level was not monotonic.

#### Training (plugged)

**Figure 6.**
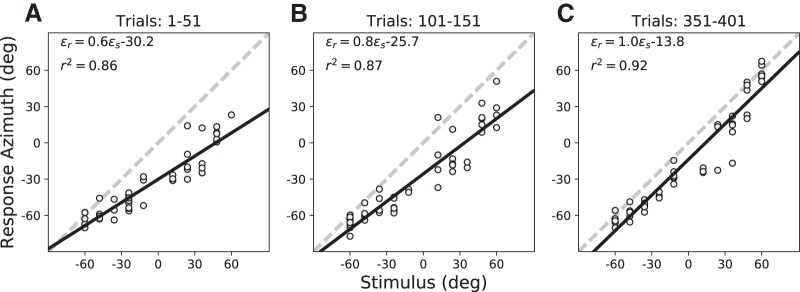
(***A***–***C***) Training phase localization data for the ten training targets (HP60 stimuli) presented in randomized order with visual feedback in the azimuth plane (elevation zero; Fig. 2) at the start of the session (trials 1–51), after 100 training trials (trials 101–151), and toward the end of the session (trials 351–401). Note the systematic increase of the response gain, and the reduction in response variability (increased *r* ^2^) and bias during the session. Data from S3.

To investigate whether explicit error feedback could improve the localization accuracy in azimuth, subjects performed a training session of ∼400 trials, in which they responded with a head-orienting saccade to one of ten selected HP60 stimulus locations in the azimuth plane. Approximately 1.5–2.5 s later, the sound was followed by presentation of a green LED at the center of the speaker, and the subject had to make a corrective head movement toward the LED, immediately after the sound-localization response. Figure 6 shows some representative sound-evoked response data from S3 for three 50-trial epochs during this session: at the start of the training (trials 1–51), after the initial phase of the training (trials 101–151), and toward the end of the training (trials 351–401). Comparing the three epochs, it can be noted that response accuracy and precision both improved as training progressed: the response gain systematically increased from *b* = 0.6 to *b* = 1.0, while at the same time the leftward bias decreased from a = –30.2° to a = –13.8°, respectively. Response precision improved as well, as evidenced by the increase in *r* ^2^.

**Figure 7.**
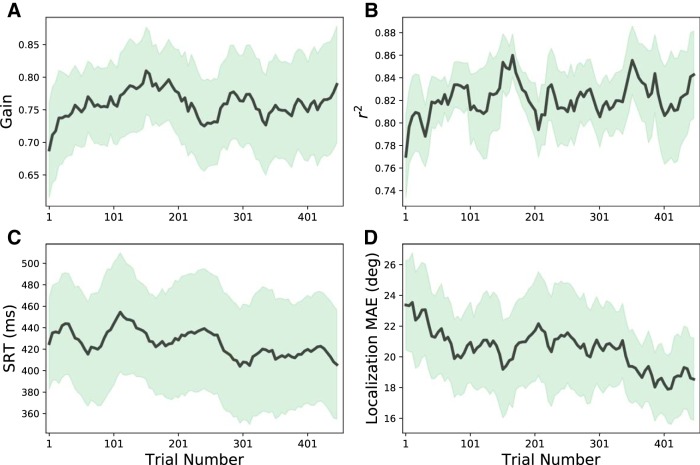
Running averages across participants (bold lines) and SD (shaded areas) of the response azimuth gain (***A***), response precision, *r* ^2^ (***B***), head-saccade reaction time (in ms; ***C***), and mean absolute localization error (in degrees; ***D***) as function of trial number during the training session. Averages of the parameters were calculated from a running-average window of 50 trials that shifted in five-trial steps through the data. Note that the response gains and *r* ^2^ values gradually increased, whereas the reaction times and localization errors to the ten stimuli decreased, which is indicative for gradually improving, and more certain response behavior during the training.

To illustrate the learning patterns for all participants during the entire training session, we performed a windowed regression analysis on the data of each listener, and averaged the results across participants. The results (mean: solid line; SD: light shading) are shown in Figure 7. The azimuth response gain (Fig. 7*A*), and localization precision (*r* ^2^; Fig. 7*B*) gradually increased with trial number, while the head-saccade reaction times (Fig. 7*C*) and the mean absolute error (MAE) across trials (Fig. 7*D*) systematically decreased. The co-variation of response variability with reaction time suggests that the auditory system becomes faster, as its confidence about perceived source locations increases.

#### Post-training (plugged)

During training, listeners had been exposed to a single stimulus type (HP60) with the right ear plugged. Sounds were presented from a limited number of only ten different locations, exclusively confined to the azimuth plane at zero elevation. Rather than true spectral-spatial learning, subjects could in principle have improved their response behavior merely by categorizing or memorizing the fixed locations on the basis of subtle acoustic peculiarities that might emanate from the speakers. If so, the improved response behavior would have persisted only for the particular trained stimulus conditions (HP60 and ten speaker locations) and would neither generalize across the two-dimensional frontal hemifield, nor to other sounds.

**Figure 8.**
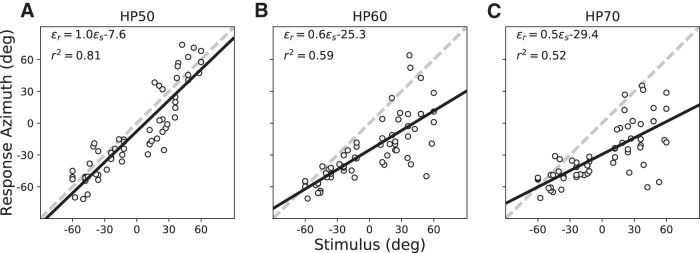
(***A***–***C***) Post-adaptation localization results for S3. Comparison of these data with Figure 5 shows that azimuth performance had improved for the non-trained azimuth-elevation locations and stimulus levels as well. Note that the leftward bias increases more systematically with increasing sound level than in the pre-adaptation tests (compare Figs. 4, 5).

To establish whether training had indeed resulted in improved sound-localization performance across the frontal hemifield, as well as for different sound levels, we re-tested the subjects after the training phase with the same three stimulus types and source locations as in the pre-adaptation session. The regression analyses (Eq. 1) for the head-orienting responses of listener S3 for these three stimuli are shown in Figure 8. The results indicate a clear improvement in localization performance, when compared with Figure 5. The response accuracy and precision for the HP50 stimuli had increased from *b* = 0.5 and *r* ^2^ = 0.69 for the pre-adaptation phase, to post-adaptation values of *b* = 1.0 and *r* ^2^ = 0.81, respectively. In addition, the response bias decreased substantially, from −36.0° to −7.6°. Thus, response adaptation was not confined to the ten trained target locations on the azimuth plane, but generalized across the two-dimensional frontal space.

When the listener was retested to these sounds after the plug was removed, localization performance was again indistinguishable from the normal-hearing control condition shown in Figure 3 (data not shown; but see Fig. 10*A*), indicating that there was no after effect of the plug or the training.

**Figure 9.**
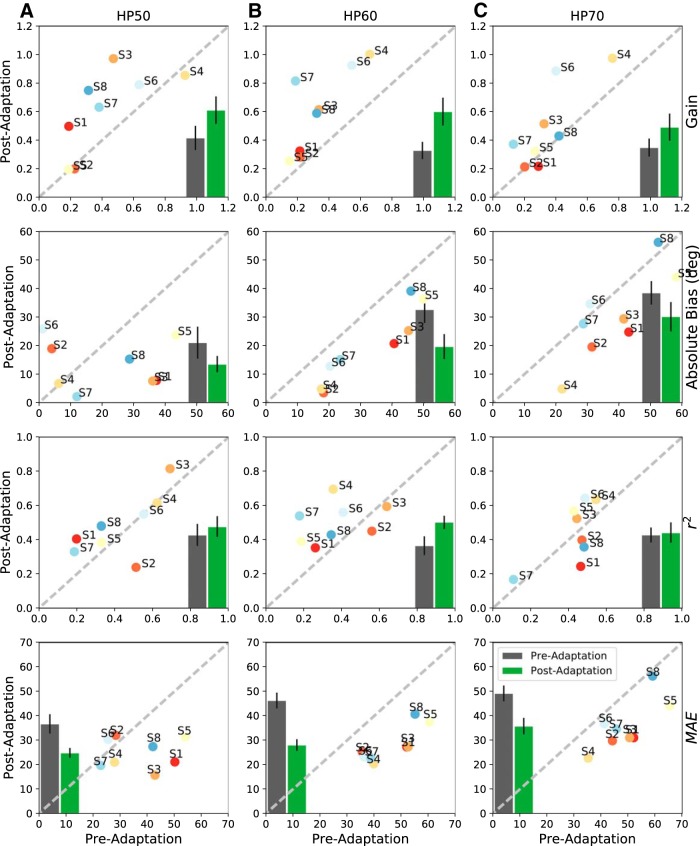
Summarized statistics of the regression analyses for all subjects for the pre-adaptation and post-adaptation tests to HP50 (***A***), HP60 (***B***), and HP70 (***C***) sounds. Columns, The three test stimuli. Top row, Response gain. 2nd row, Response bias (in degrees). 3rd row, Coefficient of determination. Bottom row, MAE (in degrees). Averages across listeners are shown as insets: gray = pre-adaptation, mean with SE, green = post-adaptation data. For nearly all four parameters and stimuli, the post-adaptation results are more accurate (higher gains, smaller bias, smaller MAE) and more precise (less variability, higher *r* ^2^).

Figure 9 summarizes the overall results for the pre-adaptation and post-adaptation tests for the HP50 (left-hand column), HP60 (center), and HP70 (right) stimuli for all listeners, together with the means and standard error of the means for the different regression parameters of Equation 1 [from top to bottom: response gain, absolute bias (in degrees), *r* ^2^, and MAE (in degrees)]. If the training had not led to improved localization performance, data points should have scattered evenly along the main diagonal, and the bars for the pre-data and post-data would have been identical. The far majority of gain and *r* ^2^ values lie above the diagonal, whereas the MAE and absolute biases lie below the diagonal. These changes in the regression parameters show a generalized improvement of localization performance for all three stimulus types and source locations.

Table 1 summarizes the significance levels of a one-sided sign test on the regression parameters (across stimuli: *n* = 24 values), and across stimulus types (*n* = 32 values).

**Table 1.** One-sided sign tests on the regression parameters across stimulus types (n/24 values) and for the three stimulus types across the regression parameters (n/32 values; see Fig. 9).

**Component**	**Gain**	**Absolute bias**	***r*^2^**	**MAE**
Azimuth	20/24 *p* < 10^–3^	20/24 *p* < 10^–3^	17/24 *p* < 0.025	22/24 *p* < 10^–4^
**Component**	**HP50**	**HP60**	**HP70**	**All**
Azimuth	25/32 *p* < 10^–3^	26/32 *p* < 10^–3^	23/32 *p* < 10^–2^	74/96 *p* < 10^–7^

n/24 signifies that n parameter values out of 24 fell above (gain, *r* ^2^) or below (bias, MAE) the main diagonal, indicative for a localization improvement. Bottom-right: all measures (n/96 values).

#### Multiple linear regression

**Figure 10.**
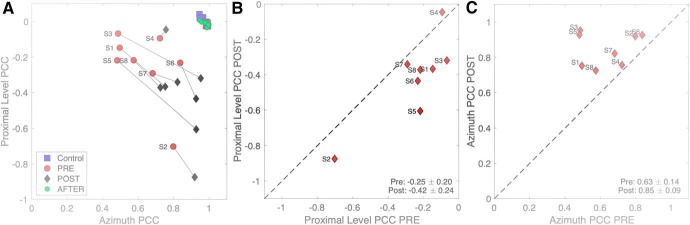
***A***, Multiple linear regression results of Equation 2 for binaural and acute monaural azimuth localization performance of each listener to sounds presented in the pre-adaptation experiments (red dots; data pooled with the plugged control data) and the post-adaptation experiment (black diamonds); *p* and *q* are the partial correlation coefficients for proximal sound intensity and target azimuth, respectively. For comparison, the normal-hearing pre-control data are also included (blue squares), as well as the results immediately after removing the plug (green dots). In these latter conditions, listeners did not rely on the HSE, as their responses were fully accounted for by the true target azimuth. Also, there was no aftereffect, as the green dots fully coincide with the blue squares. In the post-adaptation phase, the azimuth coefficient increased, while the sound-level coefficient decreased. ***B***, The change in the proximal level coefficients indicates that they decreased for nearly all listeners. ***C***, The azimuth coefficient increased for all eight listeners.

Multiple regression on the pre-adaptation and post-adaptation data according to Equation 2 assessed to what extent subjects made use of the HSE (indicated by the partial correlation coefficient for *Iprox*) and the true azimuth location, which could result from the use of monaural spectral cues, or from adjusted binaural level differences (Shinn-Cunningham et al., 1998; Van Wanrooij and Van Opstal, 2007; Strelnikov et al., 2011). Figure 10 shows the results of this analysis. The pre-adaptation plugged data for the HP50, HP60, and HP70 sounds were pooled with the plugged control data, as it contained more sound levels (Fig. 4). For comparison, we also show the results from the normal-hearing control experiment (blue squares; compare Fig. 3) and the after-effect test (green dots). Note that these latter hearing conditions yielded responses that were fully explained by target azimuth, and not at all by variations in sound level: the partial correlation coefficients for proximal sound level were indistinguishable from zero, and the azimuth partial correlation coefficients were close to 1.0.

For the pre-adaptation and post-adaptation plugged conditions, however, both partial correlation coefficients deviated substantially from the optimal normal-hearing binaural values. For the pre-adaptation data (red dots) the azimuth coefficients ranged between 0.4 and 0.8 (mean ± SEM: 0.63 ± 0.14), while sound-level coefficients ranged from –0.1 to about –0.7 (–0.25 ± 0.20). The negative values for this coefficient indicate that the louder the sound, the more leftward the azimuth response (also reflected in the large negative biases seen in Figs. 4, 5). Interestingly, in the post-adaptation data (black diamonds) both coefficients had increased (azimuth: 0.85 ± 0.09, sound level: –0.42 ± 0.24). In other words, listeners made stronger use of the HSE, as well as of the spectral cues from the hearing ear, and/or distorted binaural level differences. This conclusion is further supported by Figures 10*B*,*C*, in which the results can be seen to deviate systematically from the main diagonal for virtually all listeners.

### Elevation responses

#### Stimulus-response relation

**Figure 11.**
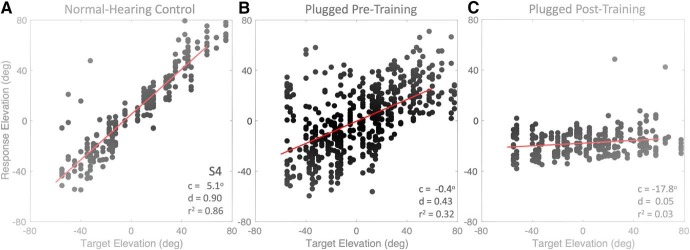
Stimulus-response relationships (Eq. 1) for elevation of listener S4 under (***A***) normal hearing, (***B***) after inserting the plug, before the training (pooled data from the control session and the HP targets), and (***C***) immediately after the training, with plugged hearing. Note the detrimental effect of the training on the listener’s elevation performance with the right-ear plug. After plug removal, the stimulus-response relation was very similar to the data in ***A*** (data not shown; but see Fig. 12*A*), indicating absence of an aftereffect.

As the extraction of source elevation relies on the pinna-related spectral cues, and training may in principle have changed the interpretation of these cues for source localization, it is of interest to test whether the training also had an effect on the elevation response components. In Figure 11, we first compared the pre-localization and post-localization data from a representative subject (S4) on the basis of the linear stimulus-response regression analysis of Equation 1. Figure 11*A* shows that listener S4 could localize the sounds well under normal hearing (apart from a few up-down reversals for downward targets, presumably due to knee-reflections), with a response gain (*d* = 0.9) close to the optimal value of 1.0, and a bias of only *c* = +5°. The variability was larger than for the control azimuth responses, but still limited, as *r* ^2^ = 0.86. The plug, however, had a strong detrimental effect on the elevation percept (Fig. 11*B*), as the pre-training data became highly variable (*r* ^2^ = 0.32), with strongly reduced accuracy: the gain decreased to *d* = 0.43 (bias: *c* = –0.4°). Interestingly, however, training seemed to induce an even further deterioration of elevation performance, rather than an improvement. After training, the post-plug results showed a much lower gain for the elevation percept (*b* = 0.05; Fig. 11*C*), the bias changed to *c* = –17.8°, and the predictability had decreased, to *r* ^2^ = 0.03. The results of the other listeners were qualitatively similar.

The decrease in elevation performance after training could in principle be due to the effects of the training on the azimuth percept (see above). For example, if training would move the azimuth percept further into the extreme left of the response range, the elevation gain, which is modulated by perceived azimuth, might become very low as a consequence. To test for this possibility, we performed two different analyses on the data: (1) one in which we quantified the local elevation gain as function of source azimuth, and (2) a multiple regression analysis, in which we incorporated other potential factors to the elevation percept than the target’s elevation angle, like source azimuth, and perceived intensity at the hearing ear.

**Figure 12.**
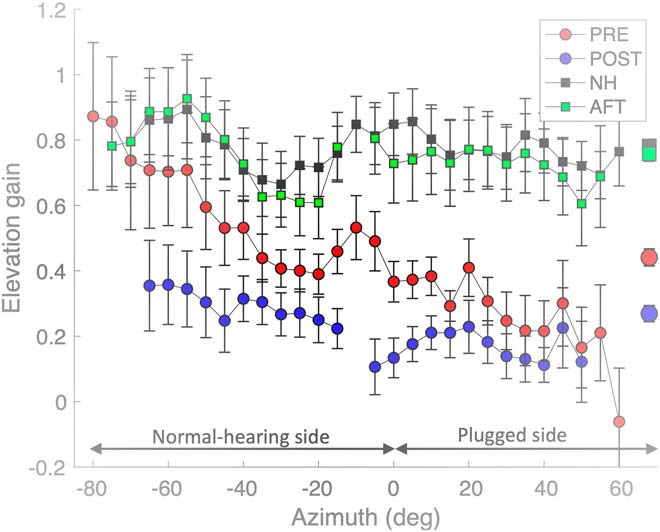
Mean local elevation gains (Eq. 1; averaged across all eight listeners, with SEs) as function of target azimuth. The local gains were determined for data selected within 20° wide azimuth bins, which were shifted in 5° steps from –80° to +60°. Note that the pre-adaptation and post-training normal-hearing control gains (black and green squares) were indistinguishable, and remained high throughout the azimuth range. The acute pre-adaptation gains (plug in right ear) show a gradual decrease of the elevation gain from normal values on the far-left hearing side to nearly zero on the far-right plugged side (red dots). After training, the mean elevation gains became very low also on the hearing side (blue dots). Symbols on the right, Overall means across azimuths and subjects.

In Figure 12, we show how the mean local elevation gains across participants varied as function of source azimuth for the four different hearing conditions. Under normal binaural hearing (black and green), the elevation gain did not vary systematically as function of azimuth, and was high throughout the target range. With the plug inserted, the acute data (red dots) reveal the typical binaural integration effect of the elevation percept (Morimoto, 2001; Hofman and Van Opstal, 2003; Van Wanrooij and Van Opstal, 2005): the gain was near-normal for targets presented on the far-left hearing side, but gradually dropped to nearly zero on the far-right plugged side. Note that targets on the midsagittal plane (at azimuth zero), had their elevation response gain at only 50% of the normal binaural gain (around 0.4, on average). Interestingly, however, and in line with Figure 11*C*, the mean elevation gains had dropped considerably on the hearing side after the training (blue dots). Although the binaural azimuth-dependent integration effect (seen in the gradual slope from left to right) was still present, it had markedly decreased when compared to the pre-adaptation data.

#### Multiple regression

**Figure 13.**
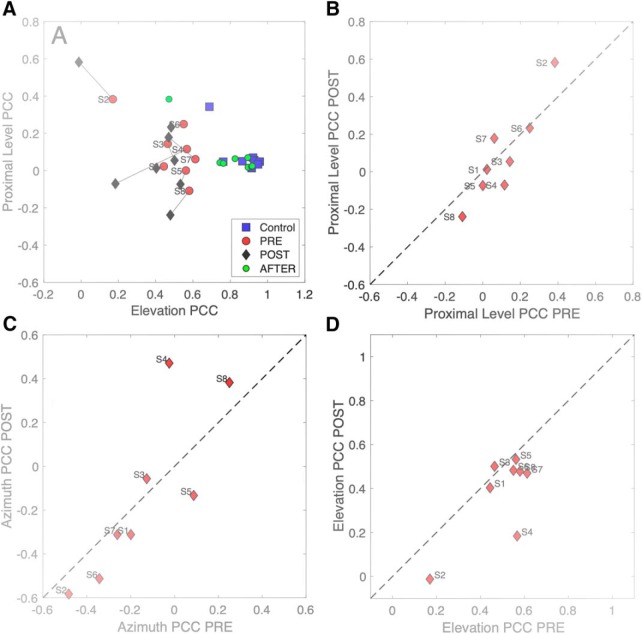
Results of multiple linear regression on the elevation response components for all listeners (Eq. 4). ***A***, ***D***, After adaptation, the contribution of the target’s elevation to the elevation response tended to decrease, whereas the contributions to the elevation percept of proximal sound level slightly decreased (***B***), but for source azimuth (***C***) did not change systematically. Note that localization performance after plug removal (green dots) was indistinguishable from the normal-hearing pre-adaptation control data (blue squares). Listener S2 had poor elevation performance in the normal-hearing and pre-plugged control, and shows up as an outlier in panels ***A***, ***B***, ***D***.

Figure 13 shows the results of the extended multiple regression analysis (Eq. 4) for all subjects. In Figure 13*A*, it can be seen (blue symbols) that the control data for 7/8 listeners (exception: S2) were close to the ideal values of *s* = 1 and *p* = 0 (*q* was close to zero too; data not shown). In the pre-plugged localization tests (control HP data and HP test data pooled; red dots) the elevation responses had a significant contribution of the true target elevation (mean: s = 0.49), and a low (near-zero) contribution of the proximal sound level (*p* = 0.11; except S2 for whom *s* remained close to zero for both epochs; Fig. 13*D*). Interestingly, the post-adaptation data (black diamonds) showed a reduction in these parameters: the contribution of target elevation dropped to a mean of *s* = 0.38 (Fig. 13*D*), while the influence of the proximal sound level stayed the same (mean 0.08; Fig. 13*B*). Yet, also the contribution of target azimuth did not change significantly across subjects (pre-mean: *q* = –0.14; post-mean of *q* = –0.13; Fig. 13*C*). Note, however, that the multiple regression was performed over the full two-dimensional frontal hemifield, and that because of the influence of the plug, elevation results could have differed for the hearing side versus the plugged side (compare Fig. 12).

**Figure 14.**
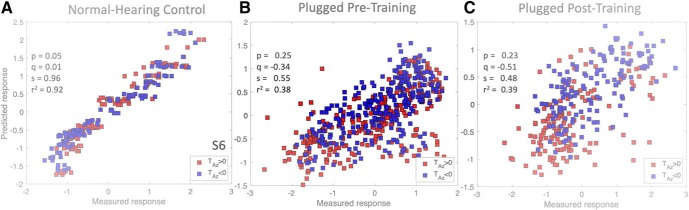
Results of multiple linear regression on the elevation responses from listener S6 for (***A***) normal-hearing, (***B***) right-ear plugged pre-training (all control data and test data pooled), (***C***) post-training data plugged. Data have been sorted for the left (hearing ear; blue) and right (plugged ear; red) side. Note the separation of upward (positive response) versus downward (negative response) perceived elevations after training for leftward versus rightward targets, respectively.

To illustrate this point for a representative listener (S6), Figure 14 shows the prediction for the elevation responses on the basis of Eq. 4 versus the measured responses for the normal-hearing control data (Fig. 14*A*), the pre-training plugged data (Fig. 14*B*), and the post-adaptation plugged data (Fig. 14*C*), expressed in normalized *z*-scores (Eq. 2). We now separated the data for stimuli presented on the hearing side (left; blue symbols) and plugged side (right; red symbols). For the normal-hearing condition, the elevation responses were equally accurate for the left- and right-side targets, as the correlation for the multiple regression model was high (*p* = +0.05, *q* = –0.01, *s* = 0.96, and *r* ^2^ = 092), and the blue and red dot distributions fully overlapped.

The elevation responses in the pre-training plugged condition (Fig. 14*B*) were much less precise on both sides (regression on all data: *p* = 0.25, *q* = –0.34, *s* = +0.5, and *r* ^2^ = 0.38), but did not differ for the left and right hemifields. However, the elevation responses under plugged hearing after the training (Fig. 14*C*) differed from the pre-adaptation responses: now the elevation responses divided in two separable clusters, in which targets presented on the plugged side (red) were typically heard at a downward elevation, whereas the leftward targets (blue) were typically heard above the horizon.

**Figure 15.**
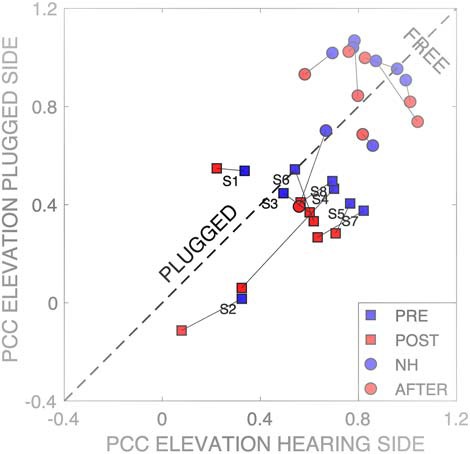
Multiple linear regression results (Eq. 4) on the elevation data for the left (hearing) versus right (plugged) hemifields for all listeners and hearing conditions. Squares correspond to the plugged conditions (blue: pre-training; red: post-training), dots indicate the normal-hearing conditions before (blue) and after (red) the training. The normal-hearing results remain unchanged, and close to the ideal value of 1.0. In the pre-training plugged condition, target elevation on the hearing side had a stronger contribution to the elevation responses than on the plugged side, as most data points lie below the diagonal. After training, responses on both sides show a decrease of the spectral cue-contributions to elevation (red squares).

To check whether the parameter changes of elevation were confined solely to one hemifield, or perhaps to both, we performed the multiple linear regression of Equation 4 separately for the left and right hearing sides. The summary of the results for the four hearing conditions for all listeners is shown in Figure 15. In the two free-hearing conditions [before (blue dots) and after (red dots) the plugged adaptation session], the elevation coefficients remained close to one and did not differ systematically for the left and right hemifields. This indicates that the training did not yield an aftereffect. In the plugged localization session before the training (in which control stimuli and HP test stimuli were pooled), the elevation coefficients were typically larger on the hearing side than on the plugged side for 6/8 subjects (blue squares, below the dashed diagonal). However, after the training, the elevation coefficients dropped substantially, and similarly, for both sides in 7/8 subjects. Thus, after training, listeners had decreased their reliance on spectral cues for localization in the elevation direction, even on their normal-hearing side (red squares).

## Discussion

### Major findings

Our experiments demonstrate short-term adaptation of sound-localization performance for all subjects in response to a monaural plug and a short training session with explicit visuomotor feedback, for a fixed-intensity HP sound source, presented at a limited number of locations in the horizontal plane. We showed that the adaptation generalized to target locations across the two-dimensional frontal hemifield, and to sounds with different intensities, indicating that the adaptation involved a remapping of available acoustic cues, rather than a mere cognitive trick imposed by the particular set of trained stimuli. The plug and training session did not invoke an aftereffect in the azimuth responses (Fig. 8*A*), or in the elevation data (Fig. 13*A*).

Interestingly, although the plug perturbed the binaural intensity differences required for azimuth localization of higher sound frequencies, the adaptation affected not only the azimuth response components, but also the elevation components. Azimuth responses became more accurate and precise after the training (Fig. 9; Table 1), but, quite surprisingly, accuracy and precision of the elevation response components deteriorated (Fig. 11), even on the unaffected free-hearing side (Figs. 12, 15).

### Ill-posed problems

To our knowledge, such differential effects of short-term training on sound-localization performance in the azimuth and elevation directions have not been reported before (but see also the section Azimuth versus elevation in acutely plugged early blind and chronic single-sided deaf). It indicates that the human sound-localization system is highly plastic, and continuously evaluates the current acoustic evidence against its internal representations. This fits well with the notion that to localize a sound, the human auditory system is in fact faced with a fundamental ill-posed problem (Middlebrooks and Green, 1991; Hofman and Van Opstal, 1998; Van Opstal, 2016): first, the ITD and ILD cues alone cannot uniquely encode sound-source direction, as all points on the so-called ‘cone of confusion’ yield identical ILD and ITD values (Blauert, 1997). Second, to disambiguate the cone of confusion, the system needs to estimate the source-elevation angle from the spectral pinna cues. However, because the sensory spectrum at the eardrum is always a convolution of the actual source spectrum and the direction-specific HRTF, both of which are a-priori unknown to the system, the extraction of elevation is ill-posed, even for a single source: infinitely many combinations of sound spectra and pinna filters (i.e., elevation angles) can generate the same sensory spectrum (Hofman and Van Opstal, 1998; Van Opstal, 2016). Third, the system should decide whether the acoustic input arose from a single source, or from multiple sources, which again poses an ill-posed problem that lacks a unique solution. Thus, on the basis of the acoustics alone, the auditory system cannot localize a sound source with absolute certainty.

To deal with this problem, the brain has to rely on additional (non-acoustic) sources of information, like visual input, priors regarding potential source locations, on the number of sources in the environment, and implicit assumptions about real-world source spectra and properties of its own pinna filters. It has been shown that the auditory system may indeed use such prior information to update its localization estimates (Hofman et al., 1998; Parise et al., 2014; Ege et al., 2018, 2019; Zonooz et al., 2018, 2019), and that it can rapidly learn to reweight its spectral contributions to the elevation percept. Experiments have also demonstrated strong plasticity to long-term changes in the spectral pinna cues (Hofman et al., 1998; Van Wanrooij and Van Opstal, 2005; Carlile et al., 2014), and in response to a visual manipulation with minifying eye-glasses (Zwiers et al., 2003). The latter indicates that visual feedback may be important in calibrating the auditory system (Zwiers et al., 2001).

### Rapid adaptation

Recently, we reported that the auditory system can demonstrate rapid short-term adaptation of localization in the midsagittal plane to repeatedly presented LP filtered noises at only six possible target locations (Zonooz et al., 2018). The results showed that listeners improved elevation response accuracy to sounds across the two-dimensional frontal hemifield, after a similarly short training session with visual feedback as in the present study. Interestingly, responses even improved without providing the visual feedback, albeit to a lesser extent. Moreover, response changes were confined to the elevation response components, and did not affect the azimuth responses. We explained these data by assuming an increased weighting of the low-frequency spectrum in HRTFs that would be associated with an increased gain (i.e., accuracy) of the localization responses, without affecting the robust binaural difference cues.

Here, we observed adjustments of localization performance to visual feedback training after monauralization. Comparison of the pre-adaptation and post-adaptation results of the multiple regression analyses indicated that the azimuth responses had an increased contribution of both the proximal sound level cue (i.e., the ambiguous HSE), and the true target azimuth (Fig. 10). The latter could be mediated by different contributions from the spectral head- and pinna cues at the hearing ear, consistent with earlier studies (Kumpik et al., 2010; Keating and King, 2013; Keating et al., 2016), and the weak, but strongly perturbed level difference cues that may have survived the strong attenuation of the plug and muff (Keating et al., 2016). If spectral pinna cues would underlie the improved performance in azimuth, also the elevation responses might have benefited from the training. However, our elevation results (Figs. 13, 14) seem to suggest that an increased use of pinna cues from the hearing ear to azimuth localization is either unlikely, or somehow interferes with the estimation process for elevation (discussed below). It is not trivial as to why the major cue for elevation (spectral pinna cues) became in fact less effective after the training, even at the normal-hearing side. We hypothesize that the improvements in azimuth were due to an increased weighting of monaural head-shadow cues (proximal sound level and LP filtered spectral cues), and of a remapping of the weak, highly perturbed, binaural level-difference cues.

Although the HSE provides ambiguous localization cues, nearly all subjects increased its contribution during training (Fig. 10*B*). This strategy may have made perfect sense, as the training was provided for a single sound level only. Although listeners were not aware of this, they learned very quickly, through the visual feedback, that the perceived sound level actually provided them with a valid cue to localize the stimulus. In the same realm, the very weak binaural difference cues that survived the plug and muff for especially the higher sound levels, could have been remapped to reduce their strong leftward localization bias, and to increase the localization gain, as observed in the data of Figure 8.

Why would the perceived elevation suffer from this brief training session? In principle, there should be no need to change the contribution of the spectral pinna cues: for azimuth, their weight is low anyway (Van Wanrooij and Van Opstal, 2004, 2007), whereas for elevation, these cues are absolutely crucial (Carlile, 2014; Blauert, 1997; Hofman et al., 1998). Our data, however, show that the elevation response gains changed by reducing the spectral elevation cues (their partial correlation was reduced by ∼23% from *s* = 0.49 to *s* = 0.38), without changing the contribution of the azimuth cues (which stayed at about *q* = –0.14) and proximal level cues (stable at about *p* = –0.10). This unexpected change in elevation behavior (Figs. 11, 12) indicates that the sound-localization system flexibly and rapidly re-weighted the different localization cues (binaural differences, spectral cues, HSE cues) and updated its internal priors, consistent with the actual acoustic situation, even if these changes would hamper daily-life hearing situations.

Indeed, during the training, the auditory system was repeatedly exposed to stimuli that provided consistent head-shadow cues (target at fixed intensity and spectrum) and binaural level differences (albeit distorted), and at the same time, source elevation never changed (i.e., remained consistently at 0°). Therefore, the adopted strategy by the listeners could have been to use the (valid) head-shadow cue, to remap the weak, but consistent azimuth cue, and to drag the mean elevation estimate toward the horizon. The latter, however, resulted in further ignoring the actual spectral cues. By emphasizing a prior assumption toward the horizon (Parise et al., 2014; next section) induced a lower gain and correlation with the actual stimulus elevation (Fig. 11*C*). The system crudely remapped sources coming from the impaired side to more downward locations, and sources from the hearing side to more upward locations (Fig. 14*C*), despite the fact that these latter stimuli contained perfectly valid pinna-related elevation cues.

### Azimuth versus elevation in acutely plugged early blind and chronic single-sided deaf

A recent study by Voss et al. (2015) on the monaural localization performance of acutely-plugged early-blind listeners demonstrated a similar negative coupling between azimuth and elevation performance than reported here for rapid adaptation in acutely-plugged sighted individuals. They grouped early blind listeners in two categories, according to their monaural azimuth performance: it either remained poor after plugging, just like in the pre-adaptation case of our sighted participants (Figs. 4, 5), or they immediately localized quite well with the plug, in which case they were shown to rely on spectral cues. Interestingly, this latter group (∼50% of their subjects) had poorer elevation performance than the former. Apparently, using spectral cues for azimuth localization (also under binaural hearing conditions in their daily lives) seemed incompatible with the use of spectral-shape cues for elevation.

In contrast, Van Wanrooij and Van Opstal (2004) described the azimuth and elevation results for chronic single-sided deaf (but normal-sighted) listeners, and showed that the more these listeners employed spectral cues for azimuth localization, the better they also localized in elevation. This suggested that spectral cues may in principle subserve both coordinates, given sufficient time (and perhaps, visual feedback). It also suggests that azimuth and elevation could rely on different, independent, but probably subtle, aspects of the HRTFs. The latter was also suggested by Voss et al. (2015).

### Mechanisms

**Figure 16.**
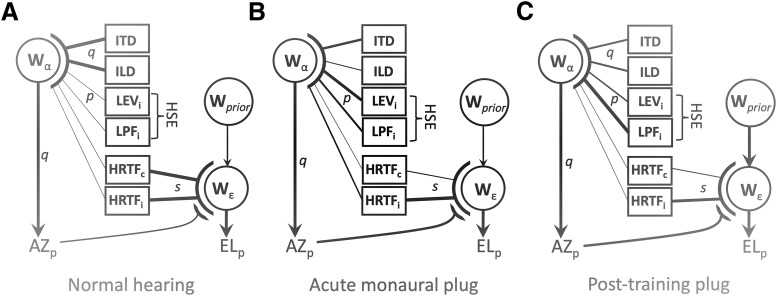
Six acoustic cues can contribute to the perceived azimuth, whereas the left- and right HRTFs determine the elevation percept, modulated by an azimuth-dependent binaural weighting and internal priors. The strength (reliability) of a cue is indicated by line thickness. p,q,s are the partial correlations, obtained from Equations 2, 4. ***A***, Under normal-hearing, the azimuth percept is fully determined by robust ITD and ILD cues, and the elevation percept mainly by the veridical HRTFs and azimuth. ***B***, After acute monaural plugging (in the contra ear, c), azimuth is determined by low-frequency ITDs, and the monaural intensity and filter cues from the HSE. The resulting azimuth percept modulates the elevation percept, thereby decreasing the weight of the plugged ear. ***C***, After training, the elevation percept is more strongly influenced by the prior (at the horizon), and less by the sensory spectral and azimuth cues.

Figure 15 extends a conceptual model (after Van Wanrooij and Van Opstal, 2007) that summarizes how the different cues are weighted to generate the azimuth and elevation percepts for the three different hearing conditions. Under normal binaural hearing (Fig. 16*A*), source azimuth is fully determined by the ILDs and ITDs, as these are the most robust and reliable cues. Elevation is specified by the monaural HRTFs of the ipsilateral and contralateral ear, whereby perceived azimuth acts as a binaural weighting factor (Humanski and Butler, 1988; Morimoto, 2001; Hofman and Van Opstal, 2003). Under acute plugging (here: contralateral ear, c), the azimuth percept loses the ILDs, as they become highly distorted and uninformative, although the ITDs may still survive for the lower frequencies. For the higher frequencies, three ipsilateral cues have increased their contribution: the overall proximal sound-level (LEV_i_), a (potential) spectral component from the LP filter of the head (LPF_i_), as well as (information derived from) the ipsilateral HRTF (Van Wanrooij and Van Opstal, 2005, 2007). After training, we observed a considerable change in the weightings for high frequencies, and a concomitant decrease of the elevation gain. The latter is not explained by a further increase of the azimuth-related cues, as a windowed analysis on the azimuth gain and bias did not show such an effect (data not shown). Our results indicate that the azimuth percept became more reliant on the (weak) ILDs and on the spectral and level HSE, whereas the HRTF cues started to contribute less to elevation. The latter percept thus fell under a stronger influence from the trained prior that the target was always near the horizon.

### Updating azimuth and elevation priors

Could a Bayesian model, in which the weights for the prior and spectral sensory cues are gradually updated, account for our results? Here, the idea would be that the auditory system assumed different, independent priors for azimuth and elevation during the different epochs of the experiment, relying on the current incoming target information (either acoustic, or otherwise). We recently suggested (Ege et al., 2018) that the normal-hearing auditory system adopts a bivariate prior for azimuth and elevation: a nearly uniform prior for azimuth (which therefore would be governed by maximum-likelihood estimation), and a Gaussian prior for elevation, centered around some default mean (Parise et al., 2014). The normal-hearing spatial prior could thus be described by

(5) $$P\left(\alpha ,\varepsilon \right)=P(\alpha )\cdot P(\varepsilon )∼\exp\left(-\frac{\alpha^{2}}{2\sigma_{\alpha }^{2}}\right)\cdot \exp\left(-\frac{{\left(\varepsilon -\varepsilon_{0}\right)}^{2}}{2\sigma_{\varepsilon }^{2}}\right),$$

in which the width of the default elevation prior may be around $\sigma_{\varepsilon }\approx$ 10–15° (Ege et al., 2018), $\sigma_{\alpha }\gg \sigma_{\varepsilon },and\;\varepsilon_{0}>0$ (upward). Under acute plugged hearing, however, the azimuth percept strongly shifts to the hearing ear, prompting a new, and narrower azimuth prior:

(6) $$P(\alpha )∼\exp\left(-\frac{{\left(\alpha -\alpha_{0}(\Delta I)\right)}^{2}}{2{\tilde{\sigma }}_{\alpha }^{2}}\right),$$

where $\alpha_{0}(\Delta I)$ is the mean of the new azimuth prior, corresponding to the perceived (plug-induced) ILD, and its new width, ${\tilde{\sigma }}_{\alpha }<\sigma_{\varepsilon }$.

During the training, visual feedback provides explicit information about the “true” target distribution, leading the listener to gradually assume that

(7) $$P\left(\alpha ,\varepsilon \right)=P(\alpha )\cdot P(\varepsilon )∼\exp\left(-\frac{\alpha^{2}}{2{\hat{\sigma }}_{\alpha }^{2}}\right)\cdot \exp\left(-\frac{\varepsilon^{2}}{2{\hat{\sigma }}_{\varepsilon }^{2}}\right),$$

with ${\hat{\sigma }}_{\varepsilon }\ll$10°, and ${\hat{\sigma }}_{\alpha }\gg {\hat{\sigma }}_{\varepsilon }.$ The consequence of the changes in these different priors is that the azimuth and elevation gains both vary with the imposed experimental conditions: the broader the prior with respect to the sensory encodings, the more the percept relies on the sensory input. Conversely, the narrower the prior, the more the percept (response) is dominated by the prior (and less by the sensory stimulus). The optimal Bayesian model [relying on the maximum-a-posteriori (MAP) response decision] is then quantified (separately for azimuth and elevation) by

(8) $$\mu_{RESP}=\frac{\sigma_{PRIOR}^{2}\cdot \mu_{STIM}+\sigma_{STIM}^{2}\cdot \mu_{PRIOR}}{\sigma_{PRIOR}^{2}+\sigma_{STIM}^{2}}\,\;and\sigma_{RESP}^{2}=\frac{\sigma_{STIM}^{2}}{{\left(1+\frac{\sigma_{STIM}^{2}}{\sigma_{PRIOR}^{2}}\right)}^{2}},$$

with $\sigma_{STIM}$ the uncertainty in the sensory input (likelihood), and $\sigma_{PRIOR}$ the width of the adopted prior. We recently provided evidence that the auditory system may in fact be suboptimal by following a different decision rule than the optimal MAP decision. In this strategy, the system aims to approximate, or match, the posterior distribution on a trial-by-trial basis by taking a random sample from the posterior (Ege et al., 2018). In this case, the response variance of Equation 8 will increase to

(9) $$\sigma_{RESP}^{2}=\frac{\sigma_{STIM}^{2}}{\left(1+\frac{\sigma_{STIM}^{2}}{\sigma_{PRIOR}^{2}}\right)}.$$

According to either model, however, the azimuth percept under normal binaural hearing can depend entirely on the acoustic input, as the binaural difference cues are highly reliable ($\sigma_{STIM}\ll \;\sigma_{PRIOR}$), so that from Equation 8: $\mu_{RESP}≅\mu_{STIM}$. The slightly more uncertain elevation percept, on the other hand, is mildly influenced by its prior, leading to a lower stimulus-response gain (i.e., $\mu_{RESP}/\mu_{STIM}$ around 0.8–0.9) than for azimuth (its gain is close to 1.0), and a small, often upward bias of a few degrees (Parise et al., 2014).

In the acute plugged condition, before feedback training, the azimuth percept becomes dominated by a new, much narrower azimuth prior ${\tilde{\sigma }}_{PRIOR}\ll \sigma_{STIM}$, leading to a low azimuth gain, and a large bias toward the hearing ear: $\mu_{RESP}≅\alpha_{0}(\Delta I)$. The elevation percept will strongly follow the influence of its prior on the impaired side (because of the low confidence for the elevation cues: low gain), but is dominated by the spectral cues on the hearing side (high gain; Fig. 12, red symbols).

During training, however, the new elevation prior (horizon, i.e., $\mu_{PRIOR}=0,{\hat{\sigma }}_{PRIOR}$ small) starts to dominate, as more evidence accumulates across trials, leading to a gradually lower response gain across the entire frontal hemifield, including at the hearing side. At the same time, the azimuth gain will increase, as it can again rely more on the (updated) sensory (spectral, and/or distorted binaural) inputs, than on the increased variance of its prior. To assess which model may better account for the data in a quantitative way, their parameters should be fitted for the different experimental conditions and results. This effort, however, falls beyond the scope of the present study.

### Visual feedback

One may wonder whether visual feedback would have been essential to induce the observed changes in localization behavior for the azimuth and elevation components. Although we have not tested this aspect in the present experiments, we conjecture that, like in a recent report on sound-source elevation (Zonooz et al., 2018), the auditory system might be able to construct a better estimate for source azimuth, merely from the repeated exposure to variations in perceived sound level, weak interaural difference cues (providing a strong bias toward the free ear), and the systematic spectral attenuation of high frequencies by the head, in combination with feedback about its own orienting movements (Hofman et al., 1998; Zwiers et al., 2001, 2003; Carlile et al., 2014). Especially the spectral attenuation by the head could provide a relatively simple and invariant monaural BB cue for source azimuth under natural hearing conditions as well, and as such serves as a valid reinforcement cue to reduce the large bias in perceived azimuth due to the plug. Note, however, that also this spectral head-shadow cue is ambiguous without prior assumptions regarding actual source spectra. However, the auditory system might infer a reasonable spectral estimate of the source from the repeated exposure to the same sound during training. It remains to be tested, however, whether the auditory system can indeed extract and combine these endogenous sources of information, and whether this would also lead to a degradation of elevation performance.

## Synthesis

Reviewing Editor: Leonard Maler, University of Ottawa Decisions are customarily a result of the Reviewing Editor and the peer reviewers coming together and discussing their recommendations until a consensus is reached. When revisions are invited, a fact-based synthesis statement explaining their decision and outlining what is needed to prepare a revision will be listed below. The following reviewer(s) agreed to reveal their identity: Andrew King, Cesare Parise.

Editorial summary

Both reviewers and the Editor agree that this is a well-designed study that extends our knowledge about how different auditory spatial cues are weighted to influence our estimates of the location of sound sources. The training paradigm is extends previous work in that the subjects (monaurally occluded participants) are given explicit visual feedback during their auditory localization training. The reviewers also made note of a second important aspect of the experimental paradigm: the ability of the participants ability to judge both azimuthal and elevation location was tested.

Both reviewers have made constructive and helpful suggestions for improvements to this manuscript and both reviewers will be asked to re-review your revisions. Please respond to the requested changes in a rebuttal letter and also in a redlined version of the manuscript.

Below, I include edited versions of the two reviews.

Reviews

Reviewer #1

How different auditory spatial cues (ITDs, ILDs, spectral cues) and other factors (e.g. sound level, head shadow effects) that influence localization judgments are integrated in the brain is an extremely interesting topic. Because these cues are - to some extent - redundant, it is equally important to know how they are weighted and whether this weighting can change with experience. Previous studies have shown that experience with a unilateral earplug results in cue reweighting, with listeners becoming more reliant on spectral cues available at the opposite ear. This work has so far focused on changes that take place in the horizontal plane.

In the present study, the sound localization performance of participants was measured within the frontal hemifield. As expected, plugging one ear degraded the accuracy and precision of the head turns toward the perceived azimuthal location of the sound. A single training session comprising around 400 trials was then provided during which participants attempted to localize highpass noise burst from one of ten locations. Clear evidence is provided that azimuthal localization performance improves over the course of these trials. Because of the fixed sound level used during training, however, this could simply be because the participants learn to associate small level differences with particular locations (which would be a trivial result). However, this is mitigated by the novel and interesting finding, based on a comparison of pre-adaptation and post-adaptation data, that improvements generalize to other sound levels and are observed when a much greater range of stimulus locations (i.e., extending beyond the training set) are considered.

The authors attempt to distinguish between the effects of azimuthal location and sound level on the localization responses, confirming previous reports that adaptation involves (at least in part) a change in sensitivity to spectral and/or binaural cues. They also examine the effects of plugging one ear on elevation responses, showing that these are degraded and then become less accurate as azimuthal localization improves. The latter result is quite striking. Through a comprehensive analysis of the data, the authors show that the impairment in elevation responses between pre- and post-adaptation testing varies with azimuth and is surprisingly pronounced on the left (open ear) side.

The authors interpret these findings as a reduction in the weighting of spectral localization cues (hence explaining the elevation results), which is associated with increased dependence on head shadow and remapping of binaural cues as the basis for the improvement in azimuthal localization. Although no direct evidence is presented for this interpretation (other than the change in response dependence on sound level), given the stimuli used during training, this seems like a plausible explanation.

Overall, this is an interesting and well conducted study, which adds to the growing evidence that the weighting of auditory localization cues is highly flexible, with implications for localization in both azimuth and elevation.

Major comments

The approach used of providing monaurally occluded participants with short-term (approx. 400 trials in one session) auditory training with explicit visual feedback is novel, as is the testing in both azimuth and elevation. However, the authors fail to cite the study by Voss et al. (2015) Trade-off in the sound localization abilities of early blind individuals between the horizontal and vertical planes. J. Neurosci. 35(15):6051-6, which reported that improvements in the use of monaural cues for localization in the azimuthal plane come at the expense of elevation performance. There are obvious key differences in that Voss and colleagues examined auditory localization behaviour in blind participants, whereas the present study looks at the effects of training on localization after plugging one ear. Nevertheless, the notion of a possible trade-off in sound localization ability between azimuth and elevation is not novel. The paper by Voss et al. should be discussed, particularly as they argue for opposing changes in the use of spectral cues in azimuth and elevation.

As far as I can tell, the loudspeaker locations used in the pre-adaptation and post-adaptation runs included those used for training. Was the difference in performance measures between the pre-adaptation and post-adaptation results still present if the training locations were excluded? This would be a stricter test for generalization to untrained locations. Similarly, do the various coefficients derived from the multiple linear regression analysis change in the same way if untrained locations only are considered?

Other comments

1. Line 6 (Abstract): “Blocking one ear will remove these [binaural] cues, and will render azimuth sound localization impossible for the listener”. This is not necessarily true since earplugs are typically low-pass filters and attenuate, rather than eliminate, inputs at the ear. Depending on the level of the stimulus used, its location and its frequency composition, the sound may still be audible at the occluded ear (as stated in the Methods), so binaural cues are distorted, not removed. Furthermore, several studies have shown that people with single-sided deafness can still localize in azimuth (in some cases, quite accurately), so it is incorrect to say that azimuth sound localization is impossible.

2. Line 15 (Abstract): “erroneous sound-localization percepts in azimuth and elevation.” This is a little confusing. Please discuss why elevation responses should be disrupted by occluding one ear (before training), as I suspect most readers would not expect this to be the case given the availability of normal spectral cues on one side.

3. Line 18 (Abstract): insert “those” before “experienced”.

4. Line 44: please specify that the frequency limit on the use of ITDs applies to humans.

5. Line 152: how “flat” were the stimuli? It would be normal practise in studies of free-field sound localization to match the output of each loudspeaker to ensure that no speaker-specific spectral cues are present. This is a concern given the comment on line 333 about “subtle acoustic peculiarities” among the speakers.

6. Line 197 onwards and 211: please specify throughout the paper which scale the presented sound levels were on (presumably dBA).

7. Line 770: “comparison of these data with Fig. 3” - you mean Fig. 5.

8. Line 802: missing ) after “targets”.

Reviewer #2

Overall, this is a carefully performed study: the experiments are methodologically sound and the analyses are also appropriate. I only have a few comments on this manuscript.

1. The authors suggest that a prior for target near the horizon may help explaining the negative effects of training on perceived elevation. This is indeed a reasonable explanation that, if tested with some modeling, would surely increase the appeal of the paper. For example, the prior could be modeled as a bivariate gaussian, with a mean specifying the location of the prior, and the variance its relative weight (in case it would be enough to just focus on the elevation and set the variance along the azimuth to infinity, see Parise et al., 2014, PNAS). During training, participants might have learnt that all stimuli come from the horizon, so that both the mean of the prior and the standard deviation got updated. If such prior is then combined with the likelihood (i.e., incoming sensory cues), the resulting localization performance should not only be biased toward the prior, but also the reported location should more tightly clustered towards the mean of the prior (i.e., the data should show less dispersion along the vertical dimension, which seems to be the case by visually comparing panels B and C of figure 10).

On a side-note, the conceptual schemes in figure 15 only show more weight being assigned to the prior, however, besides the weight (i.e., the variance of the prior distribution), participants can also learn the mean of such a prior.

2. The time course of the effects of training on localization could be more explicitly visualized by plotting the running average of the various indexes considered in the rows of figure 8 against the number of training trials. This would complement Figure 6 and highlight the temporal unfolding of perceptual learning.

3. The legend of Figure 7 invites the reader to compare figure 7 with figure 3: I guess there is a mistake here.
